# If It Is Hard, It Is Worth Doing: Engineering Radical Enzymes from Anaerobes

**DOI:** 10.1021/acs.biochem.2c00376

**Published:** 2022-09-19

**Authors:** Christof M. Jäger, Anna K. Croft

**Affiliations:** Sustainable Process Technologies Group, University of Nottingham, Nottingham NG7 2RD, United Kingdom

## Abstract

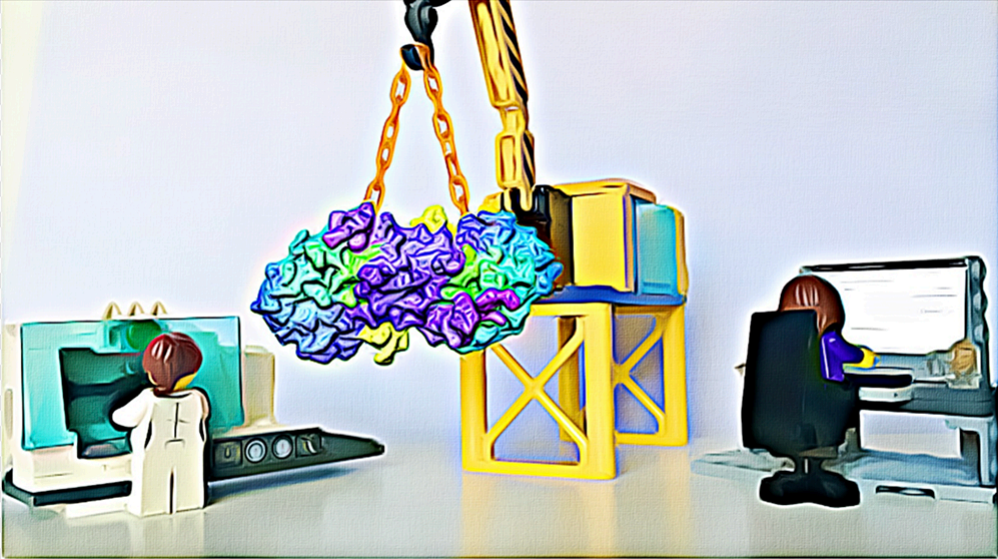

With a pressing need for sustainable chemistries, radical enzymes from anaerobes offer a shortcut for many chemical transformations and deliver highly sought-after functionalizations such as late-stage C–H functionalization, C–C bond formation, and carbon-skeleton rearrangements, among others. The challenges in handling these oxygen-sensitive enzymes are reflected in their limited industrial exploitation, despite what they may deliver. With an influx of structures and mechanistic understanding, the scope for designed radical enzymes to deliver wanted processes becomes ever closer. Combined with new advances in computational methods and workflows for these complex systems, the outlook for an increased use of radical enzymes in future processes is exciting.

## Why Radical Enzymes?

There is a recognized need within the pharmaceutical industry for efficient functionalization reactions,^1,2^ including late-stage C–H functionalization and C–C bond formation,^3,4^ that can be cleanly delivered by radical chemistry.^5^ Radical enzymes, in particular, offer a mechanism by which such transformations can potentially be sustainably embedded into synthetic industrial processes through a biotechnological approach. An added benefit is that these enzymes often already act on molecules of biochemical/medical interest, such as sugars, peptides, and nucleotides. Many of these components are precursors to a variety of antimicrobials, antineoplastics, and herbicides^6−11^ or are involved in the key metabolism of both pathogenic^12−15^ and potentially beneficial organisms,^16−18^ where analogues could be important in controlling disease.^19^ More significant is the ability of these enzymes to enact transformations that are otherwise unachievable by standard chemical routes, offering a broader range of chemistries for industrial processes (Table 1).^20^ Despite this, radical enzymes, especially those from anaerobes, are underrepresented in the protein engineering literature.

**Table 1:**
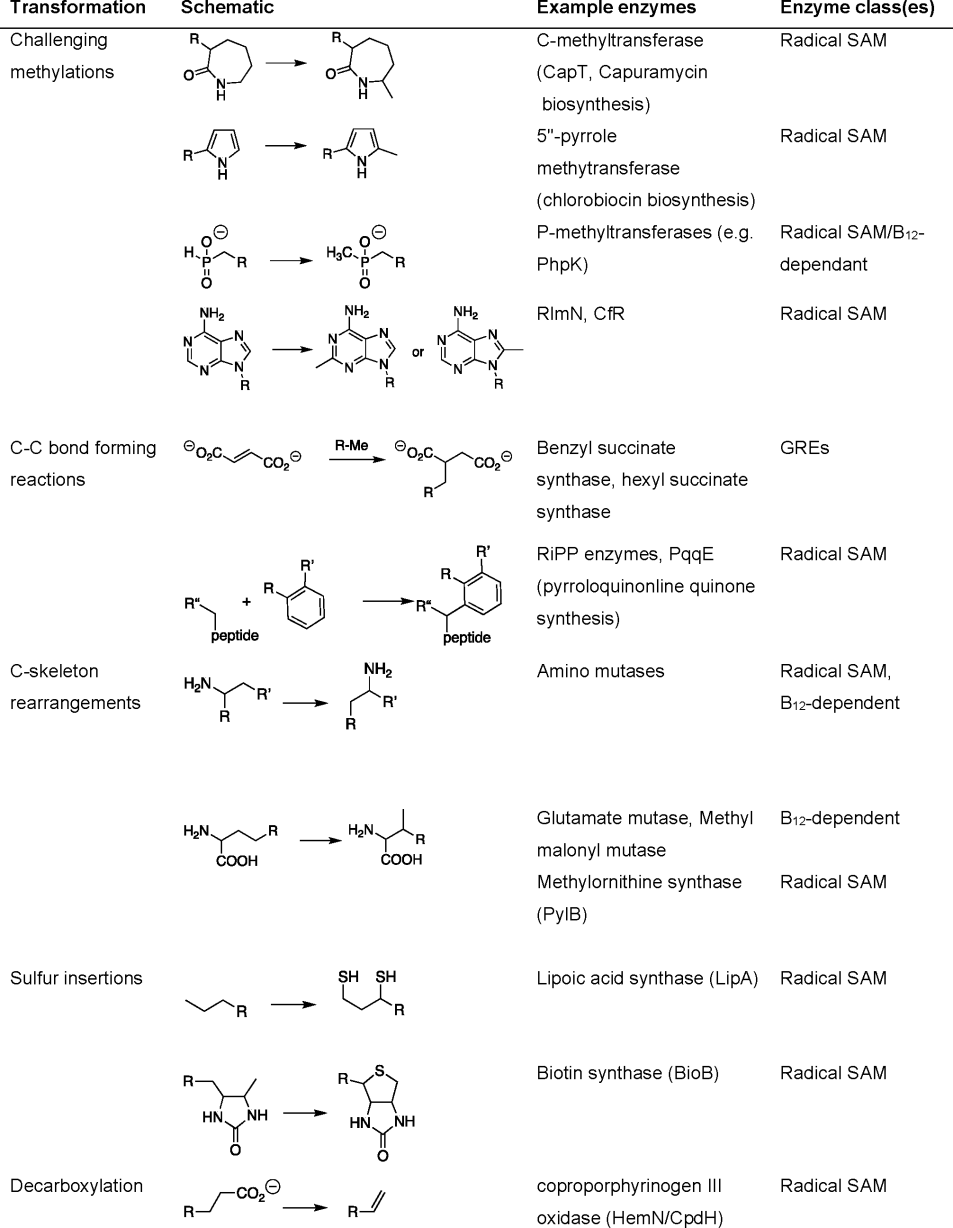

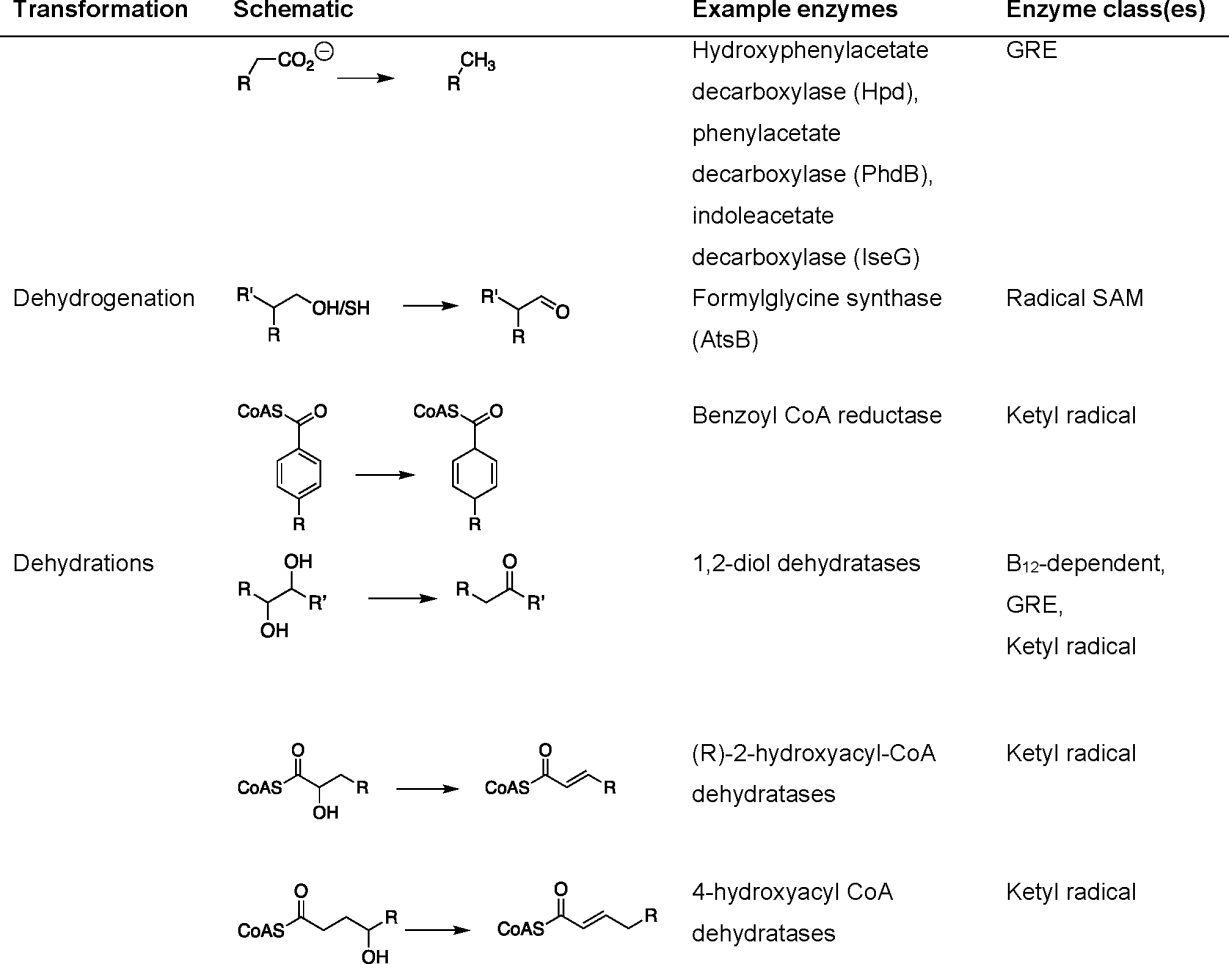
Highlighted Radical Conversions Accessible *via* Enzymes That Are Difficult or Impossible to Replicate with the Standard Polar Chemistries^a^

a The table provides selected examples by way of illustration, which are not exhaustive. The initiating radical can arise from a range of different enzymes, such as those using coenzyme B_12_, *S*-adenosyl methionine (radical SAM), glycyl radicals (GRE), iron–sulfur clusters (ketyl radical), or a combination thereof.

**Figure 1:**
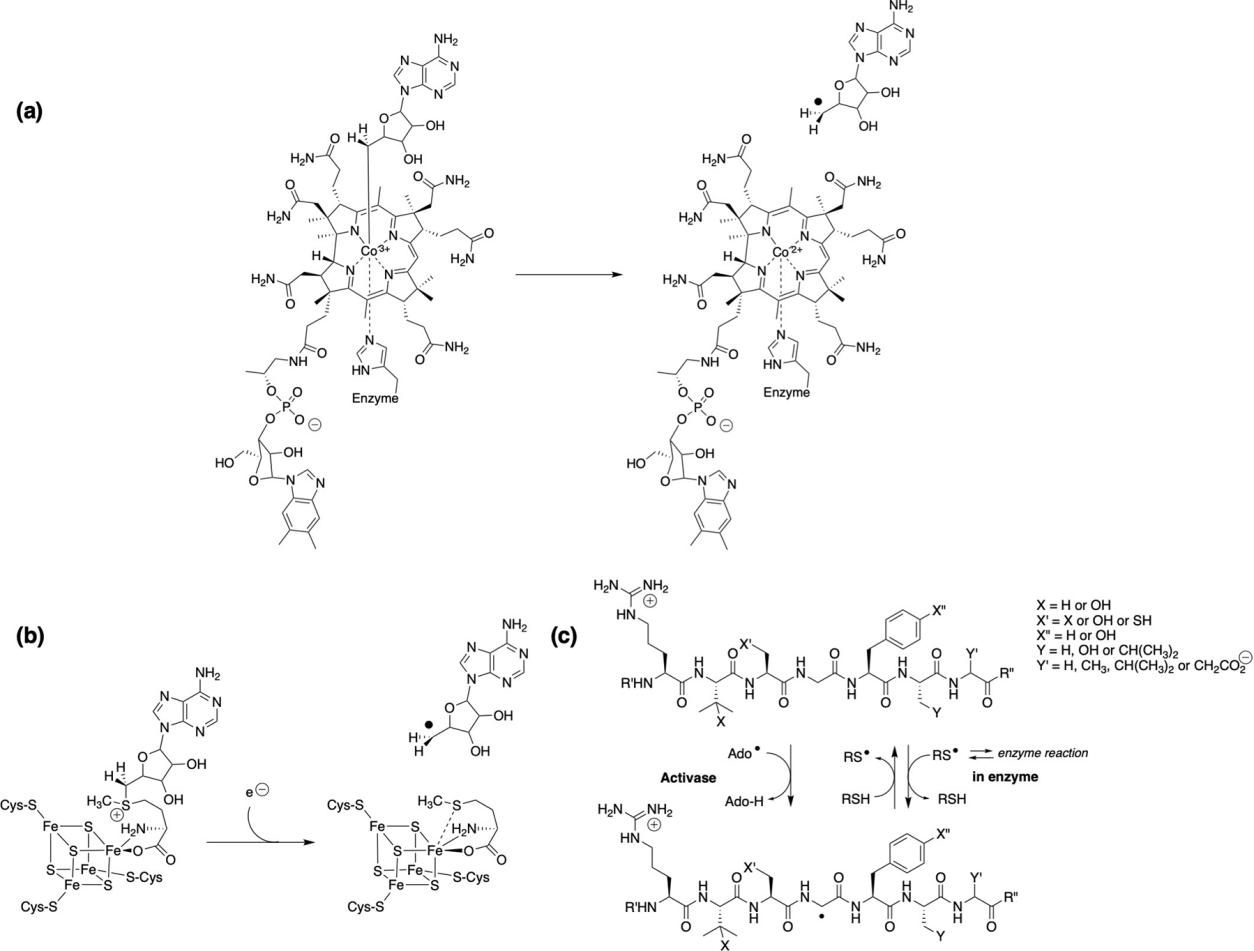
Initiation mechanisms for some classes of radical enzyme: (a) Coenzyme B_12_ undergoes homolysis of the organometallic Co–C bond to generate an adenosyl radical that reacts with the substrate; (b) *S*-adenosylmethionine-[4Fe-4S]^2+^ cleaves in canonical radical SAM enzymes to afford methionine bound to the iron sulfur cluster and the active adenosyl radical. Noncanonical cleavage has been reported for the enzyme Dph2. (c) Glycyl radical enzymes first have the backbone glycyl radical installed by a complementary activating enzyme. Once present, reaction is achieved through a relay of the backbone radical to an active site cysteine, which interacts with the substrate.

Radical enzymes from anaerobes use a variety of mechanisms to generate radical intermediates (Table 2, Figure 1). Many utilize iron–sulfur clusters, or other metallocofactors, including coenzyme B_12_,^21−23^ to generate the radical. An especially important radical initiator is *S*-adenosylmethionine (SAM) that works with an [4Fe-4S]^2+^ iron–sulfur cluster. These “radical SAM” or AdoMet radical enzymes form a superfamily catalyzing over 85 different reactions with the potential to be exploited.^24−26^ Alternatively, metalloenzymes can generate a stable protein-based radical, as in the case of the glycyl radical enzymes (GREs).^27−29^

**Table 2:** Summary of Enzymes Highlighted in This Perspective, alongside Radical Enzyme Class, Engineered Modifications

Enzyme class	Radical generation	Enzyme examples	Modification
B_12_-dependent enzyme	See Figure 1(a)	B_12_-dependent diol dehydratase (DDH)	*K. pneumoniae* S301A, Q336A, and Q336A/S301A
			*K. oxytoca* S301A, Q336A, S301A/Q336A, and Q336A/S301A/V300M
AdoMet radical enzyme (radical SAM)	See Figure 1(b)	Spore-photoproduct lyase (SPL)	*Bacillus subtilis* C141A
		Nosiheptide synthases NosL, NocL, NosN	*Streptomyces actuosus* NosL R323K, Y90A, S340A
		Lysine 2,3-aminomutases (LAM) to alanine 2,3-aminomutases (AAM)	*Bacillus subtilis* D331G and *Porphyromonas gingivalis* D339H
Glycine radical enzyme (GRE)	See Figure 1(c)	Pyruvate formate lyase (PFL)	Fusion protein formation
**Other classes**			
B_12_-AdoMet enzymes	Primarily as per AdoMet radical enzymes	*e.g.*, OxsB involved in oxetanocin A biosynthesis	
Ketyl radical enzymes	One electron transfer ([4Fe-4S] or [4Fe-4S]/FAD)	*e.g.*, (*R*)-2-Hydroxyacyl CoA dehydratases (“archerase”), benzoyl-CoA reductase	

Both B_12_-dependent and AdoMet radical enzymes generate an adenosyl radical intermediate (Table 2).^21,30^ In B_12_-dependent enzymes, the homolytic cleavage of the adenosyl unit from the cobalt of the corrin ring is induced by changes in enzyme structure. In contrast, the adenosyl radical in AdoMet radical enzymes must be generated through initial reduction and cleavage of the AdoMet-[4Fe-4S]^2+^ iron–sulfur cluster, often induced by flavodoxin.^30^ In GREs, the protein radical is post-translationally generated by an AdoMet enzyme activase, with the substrate radical formed by transfer of the backbone glycyl radical to an active site cysteine, which then reacts with the substrate.^28^ This latter transfer is mediated by substrate-specific enzyme contacts, on binding of the substrate.

Radical generation on a substrate, by a process of single electron transfer, can also be directly carried out through inorganic species, such as iron–sulfur clusters (ferredoxin or flavodoxin), sometimes with cofactors such as FAD or with activation by ATP to generate the needed low reduction potentials. Examples here particularly include those processes generating ketyl radical intermediates.^31^

## What Are the Challenges?

The high reactivity of radicals has presented challenges to the development of these enzymes for industrial purposes. For aerobically sensitive enzymes, specialist equipment and techniques are often required to both characterize the enzyme mechanisms or generate the appropriate crystal structures needed to fully enable rational engineering approaches. This oxygen sensitivity and enzyme cofactor/cosubstrate requirements are also seen as potential limitations to industrial use by some, although in vivo use of some radical enzymes has been shown to be practical.^20,32^ The slow rates of radical enzymes, such as biotin synthase (BioB) and lipoic acid synthase (LipA), could also pose a bottleneck for scale-up; however, this argues a case more strongly for exploring these limits through protein engineering methods.

At the molecular scale, many of these enzymes carefully limit access to the active site and/or bind the substrates in a very specific orientation. These control mechanisms are needed to direct the reactions in the face of what can otherwise be an unspecific reaction, driven by the high energy of the initiating radical. Active site access thus either needs to be preserved to prevent side reactions and cofactor inactivation or selectively engineered to leverage this as an opportunity to incorporate additional functionality, but often at the expense of the reversibility of any radical process. Similarly, when undertaking an engineering process to generate an intermediate radical at either a different position on the natural substrate or on an entirely new substrate, binding interactions and the flexibility of the substrate in the active site need to be carefully considered to ensure the desired product is obtained. Any specific substrate–protein interactions triggering radical initiation upon substrate binding will still also be needed. Finally, the substructures that bind and stabilize the metallocofactors, cosubstrates, and/or radical units need to be approached with care to retain and not destroy the key functionality, limiting changes that might be made. The scope of changes accessible here can be informed by the extensive bioinformatic information available now for radical enzymes.

More broadly, many radical enzymes need to be either activated, reactivated, or have auxiliary units replaced as part of the unusual reactions catalyzed (*e.g.*, sulfur insertions). This adds a further level of complexity to maintain the protein–protein recognition elements needed for repair, which are not yet understood in detail. Nevertheless, some progress has been made to successfully engineer radical enzymes despite these challenges and point the way to future approaches.

## Direct Manipulation of Specific Residues

**Figure 2:**
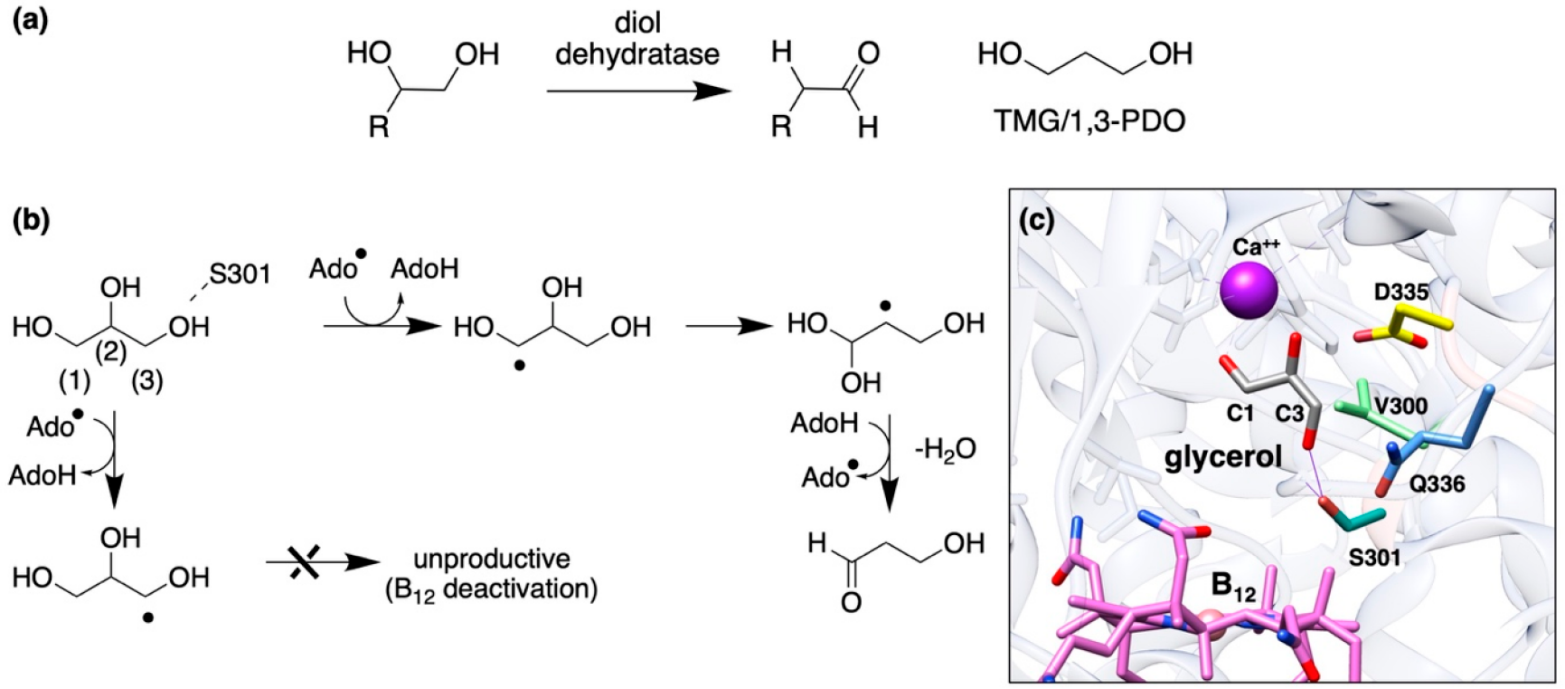
(a) Generic reaction scheme for diol and glycerol dehydratases, which are important in the industrial production of TMG. (b) Outline mechanism for glycerol dehydration *via* the B12-dependent diol dehydratase (DDH). Binding in the pro-(*R*) form favors hydrogen abstraction from C1 to generate the product aldehyde. In contrast, when binding in the pro-(*S*) form, competitive abstraction from either C1 or C3 can occur, with C3 abstraction leading to a dead-end, stable intermediate radical, and subsequent inactivation.^39^ (c) The active site of DDH from crystal structure 3AUJ, showing bound glycerol with a hydrogen bond to S301, alongside complexation to Ca^2+^ and cyanocobalamin (B_12_) in place of the cofactor adenosyl cobalamin. Mutants explored for improved activity, S301, Q336, and V300, are shown. When adenosyl cobalamin is bound, D335 preferentially (but not exclusively) replaces S301 in hydrogen bonding and orienting the C3 OH.^38^

Mutational studies have primarily been carried out to elucidate key mechanistic points^33^ but can also be seen as entries into expanding and exploiting the substrate scope for many radical enzymes. Because of the industrial relevance of the B_12_-dependent diol dehydratase in the production of the major polymer precursor trimethylene glycol (TMG, 1,3-PDO, Figure 2a),^34^ there have been various attempts at engineering both this protein and the bacterial chassis producing it.^32,35,36^ A key challenge has been the glycerol-induced inactivation of the B_12_-cofactor (Figure 2b), and mechanistic information, alongside X-ray crystal data (Figure 2c), has been fundamental to redesign. Glycerol, a prochiral molecule, is able to bind in two forms: one that reacts to form product (pro-*R*) and the other (pro-*S*) that results in an irreversible cleavage of the cofactor C–Co bond.^37−39^

**Figure 3:**
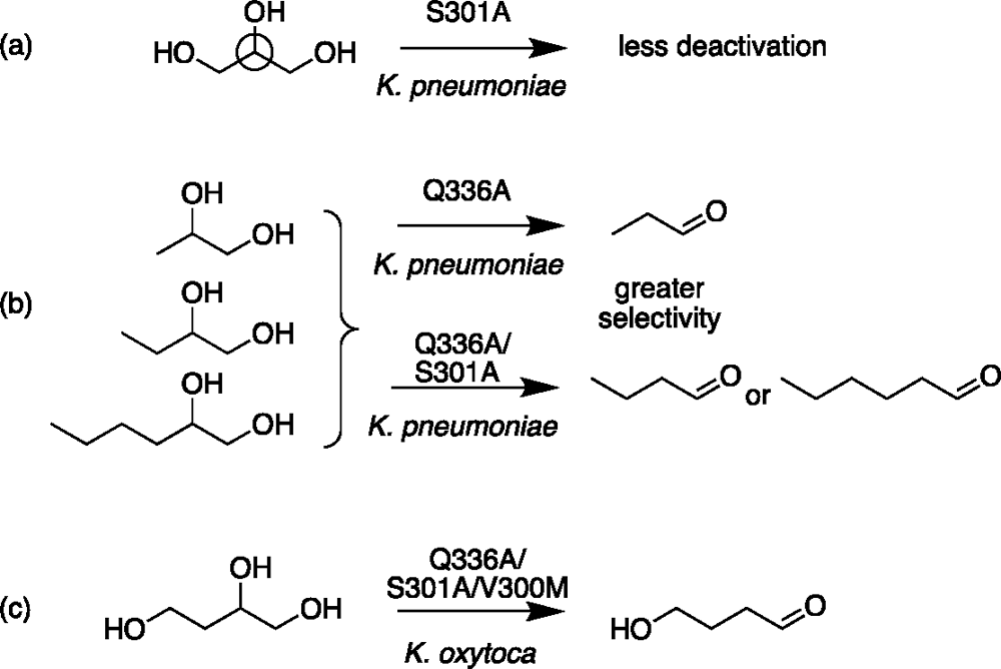
Impact of bacterial dehydratase mutations on product formation. (a) The prochiral center (circled) has an impact on dehydratase inactivation, which can be reduced with the S301A mutant of the *K. pneumoniae* diol dehydratase. (b) Selectivity for 1,2-PD vs 1,2-BD and 1,2-HD is increased for the Q336A mutant, whereas the Q336A/S301A mutant shows improved selectivity for longer-chain species. (c) The double mutant Q336A/S301A accepts 1,2,4-butanetriol as a substrate, with further improved activity for the Q336A/S301A/V300M triple mutant.

Replacing S301 in the *Klebsiella pneumoniae* diol dehydratase, a residue potentially making a critical hydrogen bond with the 3-OH of glycerol, with alanine afforded a mutant that was ∼2.7 fold less prone to deactivation by glycerol than was observed for the wild-type enzyme (Figure 3a).^35^ In addition, both this and a mutant that disrupted the nearby protein backbone hydrogen-bonding network, Q336A, gave improved selectivity to 1,2-propanediol (1,2-PD) over the longer-chained 1,2-butanediol and 1,2-hexanediol (Figure 3b). In contrast, combining these two mutants resulted in much better activity against the longer-chain diols (Figure 3c), which was also reflected in the crystal structure by a larger space to accommodate the larger alkyl groups.

Similarly, the *Klebsiella oxytoca* diol dehydratase has been rationally engineered to process 1,2,4-butanetriol (1,2,4-BTO) as a substrate, with 5-fold higher activity than the corresponding wild-type enzyme.^36^ In combination with engineered improvements to *E. coli* xylose catabolism, the industrially important 1,4-butanediol (1,4-BDO) could be produced in yields of up to 209 mg/L. Here a fusion dehydratase linking the three subunits was selected as the starting point, which had already shown improved activity toward the native substrates for the production of 1,3-PD and 1-propanol.^40^ When feeding studies of 1,2,4-BTO did not result in production of 1,4-BDO, a rational design approach was used to engineer the appropriate activity. The same S301A and Q336A mutants as previously reported for *K. pneumonia*(35) resulted in decreased inhibition and increased production of 1,3-PD, with the double mutant S301A/Q336A showing ∼4.4 fold improvement in 1,4-BDO producing activity. By considering the key interaction of the diol with coordinating potassium in the active site, three candidate residues were identified for mutagenesis where this coordination might become more favorable, specifically T222, V300, and F374. In silico screening *via* substrate docking and subsequent testing in vitro provided maximum activity from a combined S301A/Q336A/V300M mutant, with ∼5-fold overall improvement over wild-type activity against 1,2,4-BTO as substrate.

Dehydratases with improved reaction kinetics have also been engineered using error-prone PCR and high-throughput screening, showing that these methods can be effective for radical enzymes.^41^ There is still more scope in dehydratase engineering, and this is being coupled with extensive metabolic engineering to achieve ever more effective production of industrially important chemicals.^32^ The higher atom economies and lower production costs establish these bioprocesses as effective alternatives to fossil-fuel conversions.

**Figure 4:**
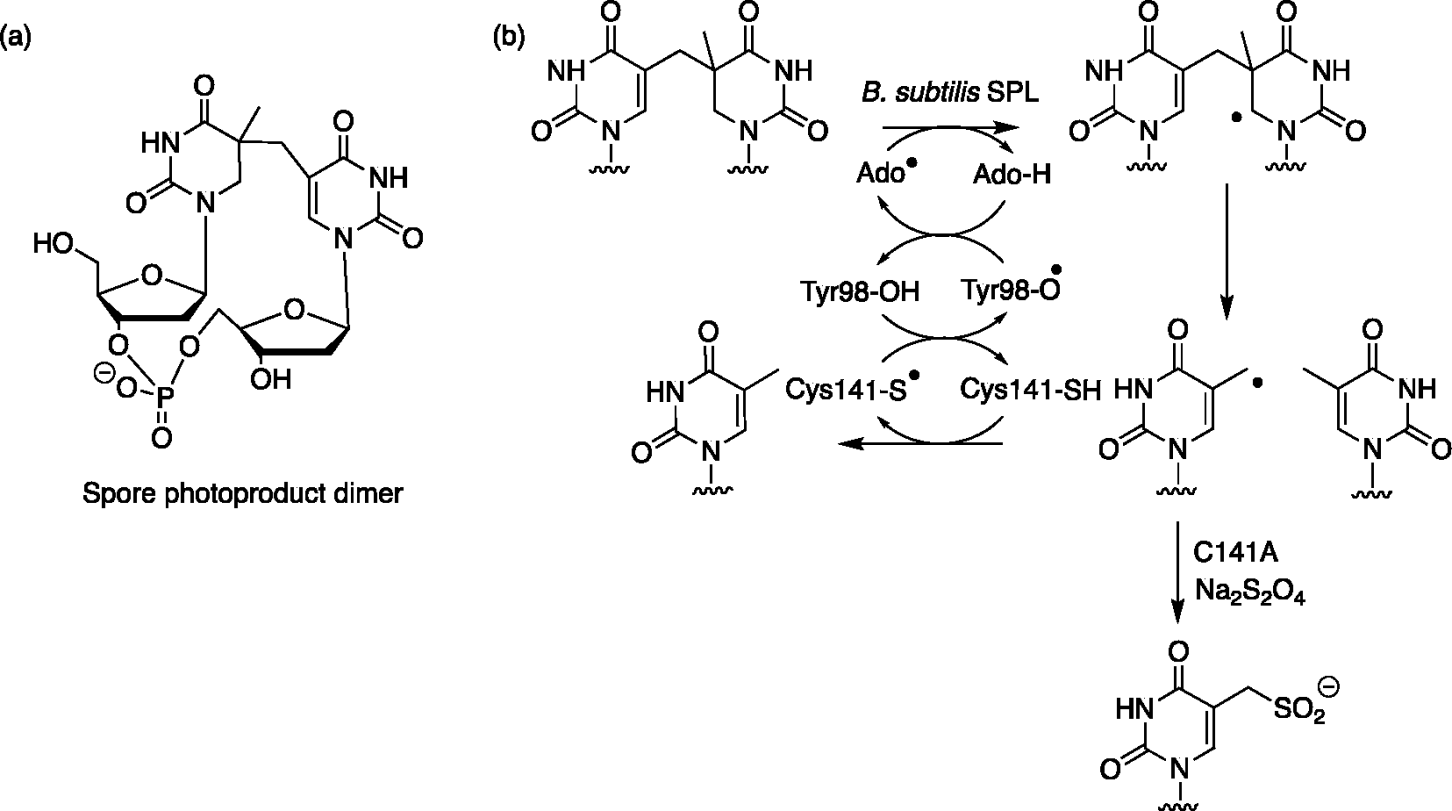
(a) Structure of the spore photoproduct dimer model system. (b) Proposed mechanism of the *B. subtilis* spore photoproduct lyase (SPL).^43^ Hydrogen abstraction is followed by dispropotionation to separate the thiamine dimer motif. Under normal circumstances, it is proposed that Cys141 donates a hydrogen atom, with a subsequent radical cascade to reactivate AdoH. In contrast, the mutant C141A cannot quench the product in this way and in the presence of sodium dithionite affords the corresponding sulfinated product.^42^

One important role of the scaffolding in radical enzymes is not only to prevent cofactor inactivation, as outlined above, but to prevent reaction of the highly reactive intermediates with anything other than the substrate. In engineering terms, modifications that disrupt the control enabled by this scaffolding can be exploited to generate new products. The single site-specific mutation of C141A in the AdoMet radical enzyme *Bacillus subtilis* spore-photoproduct lyase (SPL) highlights the ease of access to divergent products within radical reactions (Figure 4).^42^ By removing the hydrogen atom donor for the last step in the mechanism (Figure 4b),^43−45^ a >90% yield of sulfinated thymine derivative could be obtained.^43^ This outcome suggests that release of the tight control that radical enzymes have over their reactions could be exploited in creating alternative products.

**Figure 5:**
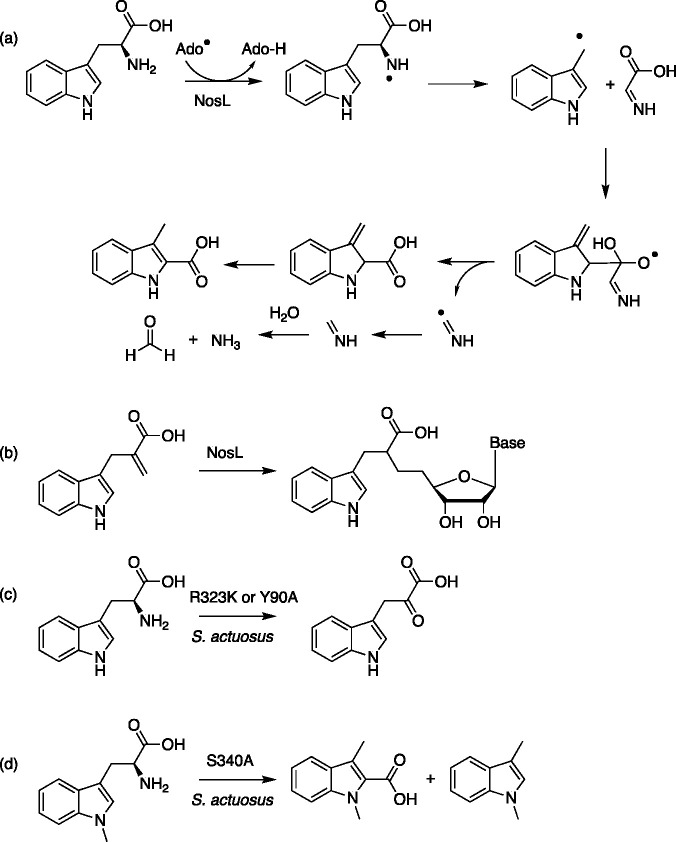
(a) The outline mechanistic proposal for the reaction of tryptophan with NosL to generate 3-methyl-2-indolic acid, formaldehyde, and ammonia.^46,48,52^ (b) Olefins can react to trap the intermediate Ado· radicals (or Gua/Cyt radicals from the corresponding derivatives) and generate nucleoside-based products. (c) The *S. actuosus* NosL mutants R323K and Y90A are both able to form indole-3-pyruvic acid. (d) The alternate substrate *N*_1_-methyl-l-tryptophan generates a mixture of 1,3-dimethyl-1*H*-indole and 1,3-dimethyl-1*H*-indole in the presence of the *S. actuosus* NosL mutant S340A.

The high reactivity of radicals means more direct active site modifications can lead to a range of different reactions, complementing the substrate promiscuity already seen in some radical enzymes. The nosiheptide (and analogous) synthetic pathways are part of the class of ribosomally synthesized and post-translationally modified peptide (RiPP) pathways.^8^ These pathways show significant scope for the creation of new antibiotic variants^46^ and integrate AdoMet radical enzymes NosL, NocL, and NosN. NosL has been heavily characterized and catalyzes the reaction of tryptophan to afford 3-methyl-2-indolic acid, formaldehyde, and ammonia (Figure 5a). Substrate analogues revealed cryptic reaction modes for NosL^46−48^ and suggest that engineering efforts to constrain the substrate into specific orientations may be effective for directing specific reaction outcomes. Similarly, reaction of NosL with an olefin substrate analogue and SAM nucleoside analogues (Figure 5b), where the adenosine is replaced by guanine and cytosine, highlights scope in generating nucleoside products more effectively by engineering the adenosine recognition sequence.^49^ Although these broad substrate scopes were the result of natural enzyme promiscuity,^50^ synthesis of indole-3-pyruvic acid (Figure 5c) instead of 3-methyl-2-indolic acid (Figure 5a) could be induced by the R323K mutant of *Streptomyces actuosus* NosL, albeit at lower activity than wildtype reactions.^48^ The same product was also observed in reactions of the Y90A variant.^51^ The corresponding S340A mutant was able to accept the non-wild-type accessible substrate *N*_1_-methyl-L-tryptophan to afford a mixture of 1,3-dimethyl-1*H*-indole and 1,3-dimethyl-1*H*-indole (Figure 5d).^48^ Modified nucleosides were also accessed through the R323K mutant through reaction with indole-3-pyruvic acid.^51^

**Figure 6:**
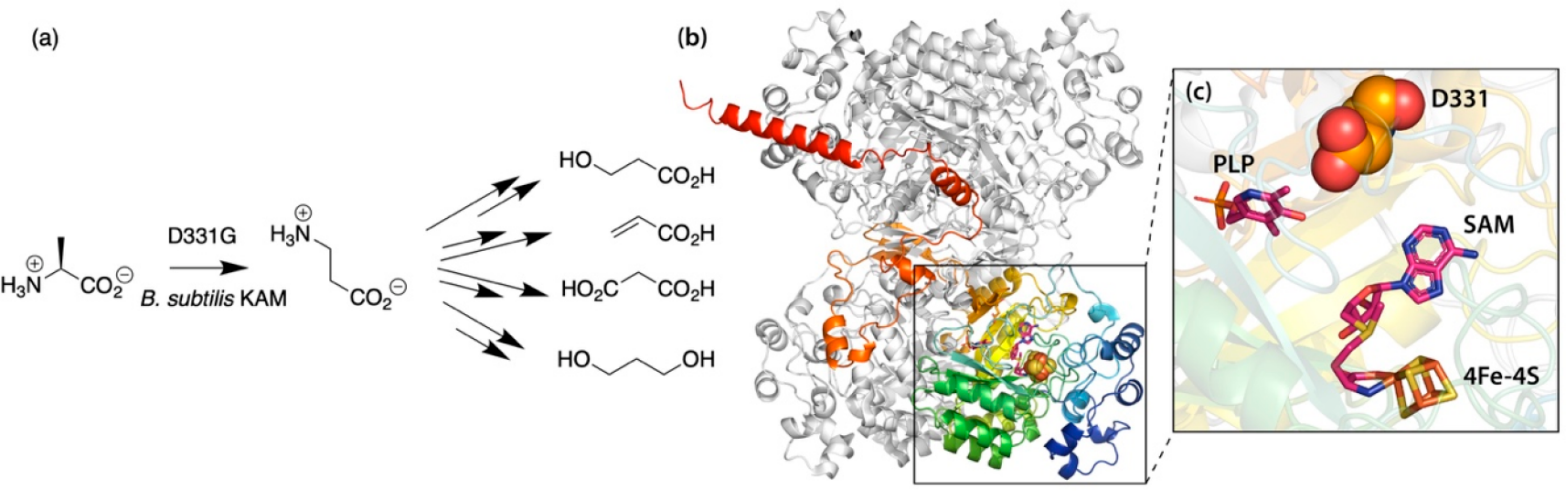
(a) Reaction catalyzed by alanine 2,3-aminomutase, created from mutants of either *B. subtilis* or *P. gingivalis* lysine 2,3-aminomutase, leading to a range of industrially useful downstream products. (b) Tetramer of 2A5H (*Clostridia subterminale*, gray) overlaid with the single subunit *B. subtilis* lysine 2,3-aminomutase (uniprot: O34676, rainbow) created by Alphafold2.^55,56^ (c) The active site of *B. subtilis* lysine 2,3-aminomutase. The key residue D331 is indicated as orange spheres with red oxygen atoms between the cofactor PLP (left) and [4Fe-4S]^2+^·SAM (below, right).

A similar broadening of substrate scope can be accessed through library-based approaches. Amino mutases for various transformations are extremely valuable, and their products provide routes to desirable chemicals, such as 3-hydroxypropionic acid, acrylic acid, malonic acid, 1,3-propanediol, and many others (Figure 6a). Cargill patented enzymes possessing alanine 2,3-aminomutase activity that had been derived from a mutagenized library of *Bacillus subtilis* and *Porphyromonas gingivalis* lysine 2,3-aminomutases, both AdoMet radical enzymes.^53^ The key and common mutation identified was D331G (*B. subtilis*) and D339H (*P. gingivalis*), although a number of mutations were generated through the library approach. Homology modeling^54^ and structure prediction^55,56^ (Figure 6b) locate this change as an aspartate in the active site between PLP (pyridoxal phosphate) and SAM (Figure 6c). In addition to a direct role, mutation may result in altered alignment of the Ado· radical or possibly changes in reactivity induced by modified electrostatics, similar to the proposed impact of residues in B_12_-dependent enzymes.^57^

The examples presented above demonstrate how single site-specific mutations can be harnessed to change reaction outcomes of radical-bearing enzymes, often through disruption of single hydrogen bonds or hydrogen bond networks. The examples show changes in substrate scope and product specificity and already offer opportunities for new antibiotics and enhancements to industrial production. Importantly, they indicate that a judicious and rational selection of even single residues can make a difference that does not necessarily destroy the careful control that radical enzymes have over their substrates and that these changes are within range for immediate, calculated improvements to existing characterized enzymes.

## Protein–Protein Interactions

**Figure 7:**
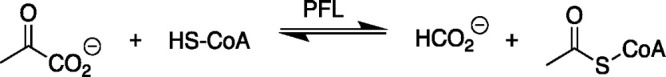
Pyruvate formate lyase (PFL) catalyzes the reversible conversion of pyruvate to formate.

Given the challenges of developing efficient bioprocesses, creative and multifaceted enzyme engineering approaches are needed. Such approaches are exemplified by the recent development of a pyruvate-production system with modified pyruvate-formate lyase (PFL).^58^ PFL is a glycyl radical enzyme (GRE) that catalyzes the formation of formate and acetate from pyruvate (Figure 7)^59−62^ but can be used in the reverse direction to generate the useful biosynthetic precursor pyruvate from assimilation of the readily accessible C1 molecule formate. Compartmentalization, which has been shown to help protect anaerobically functioning systems from oxygen,^63,64^ has been exploited to achieve this production system. Protein engineering featured to achieve appropriate functionality.^58^ A fusion protein of PFL with a phosphotransacetylase (EutD) was formed with a glycine/serine linker. This fusion ensured that the PFL substrate acetyl CoA (formed from CoA and acetyl phosphate by EutD catalysis) could be produced in the close vicinity of PFL to reduce mass transfer limitations. Tags (SpyCatcher) were added to the PFL-EutD fusion protein as well as PFL, *via* either one of the internal loop regions or the *C*/*N*-terminal regions. These tags allowed integration into a protein-based supramolecular protective shell (based on bacterial microcompartments (BMCs)) by complementary binding to a “SpyTag” motif. Results showed that the PFL-EutD fusion had low expression, and the *N*-terminal and insertion fusions of the SpyTag with PFL were insufficiently functional. Thus, a *C*-terminal SpyCatcher-adapted PFL was selected for synthetic BMC binding, and mass transfer to EutD was instead enhanced through an orthogonal SnoopCatcher/SnoopTag system to integrate this enzyme also within the synthetic BMC. The fully assembled BMC “wiffleball” demonstrated a PFL *k*_cat_ of ∼1.2 s^–1^ and an estimated 1000 turnovers per enzyme until activity was lost, showing that the anaerobic chemistry was able to proceed under aerobic conditions once protected.

The broader interactome network has been shown to have a significant impact on especially iron–sulfur cluster enzymes, and thus, engineering of these related enzymes impacts the success of these processes in in vivo systems. Mutants of the repair protein IscR have exhibited significantly improved biosynthetic activity for a number of pathways involving radical enzymes,^65^ primarily through mitigating the depletion of FeS clusters caused by radical enzyme overexpression. As a result, either incorporation of these mutants or, alternatively, complete deletion of the IscR gene is now becoming a standard technique for in vivo production improvement.^65−69^

## Computational Approaches to Screening

**Figure 8:**
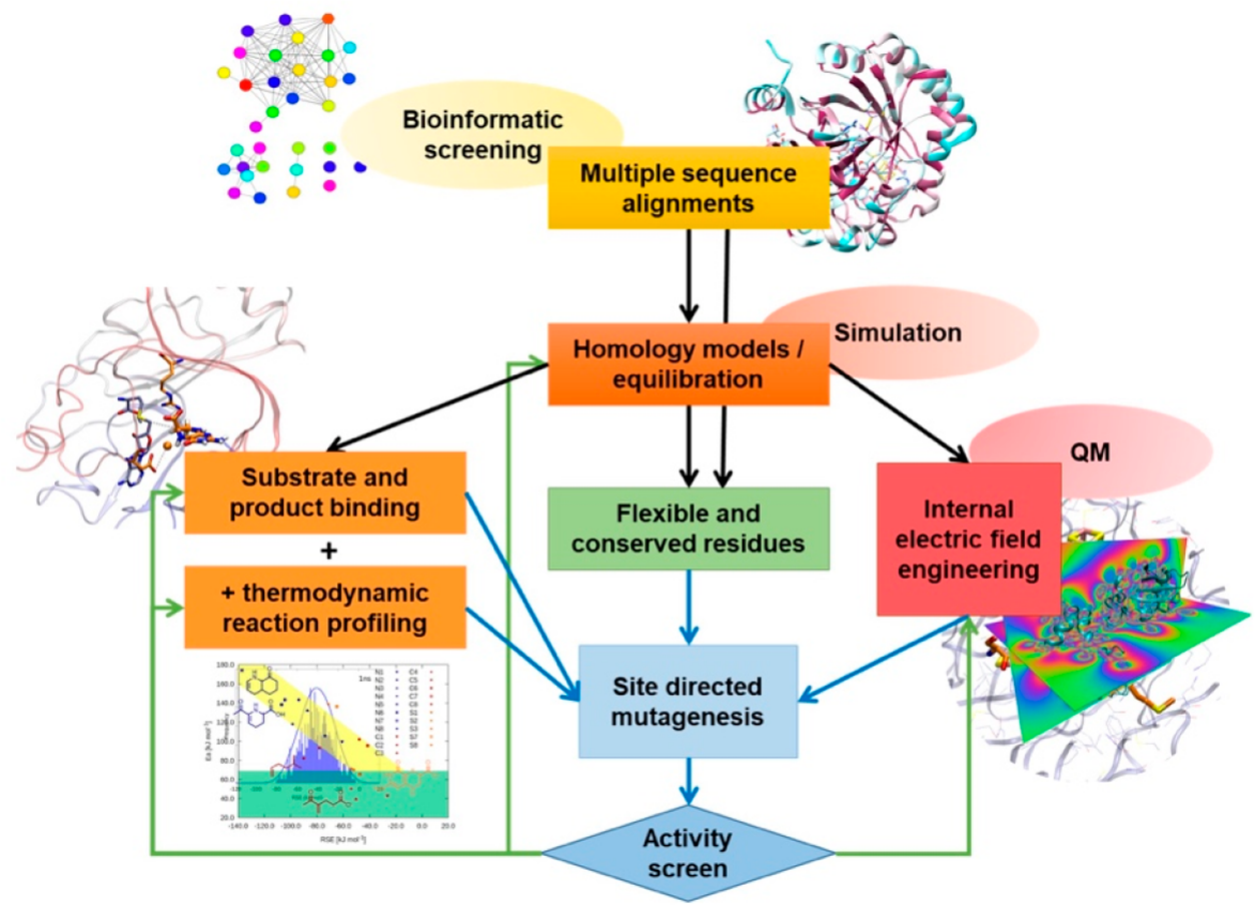
Example workflow for integrating thermodynamic and electric-field screening to identify and validate efficient mutants of radical enzymes.

One way to interrogate and circumvent the rapid reactivity of radical intermediates when carrying out experiments, and to account for the all-important aspect of protein dynamics, is to consider these species in silico—*i.e.*, through computational modeling.^70^ Such approaches lend themselves to screening, with a rapid set of modifications being enabled and tested (Figure 8). An important factor is how to and what to screen these systems for. The detail of radical chemistry is best captured by the computationally expensive quantum mechanical (QM) methods because they treat the electronic interactions. Indeed, model systems for many radical enzymes have been used with success to understand mechanistic elements. Calculations on radical systems, however, are not always straightforward and require particular care, due to the nature of the unpaired spin.

The role of the enzyme superstructure in influencing the active site chemistry is increasingly recognized as being able to significantly influence the outcome, not least through a steric and an electronic perspective. Where this more complete picture is needed, the large size of proteins usually requires molecular dynamics (MD) to be employed. With better access to a computational resource, combined QM-MM methods have been successful in establishing the details of some radical mechanisms.^71−80^ Such studies are important in establishing the core residues and enzyme features important for controlling radical reactivity and selectivity and thus identifying targets for later protein engineering efforts.

Alternatively, less computationally expensive approaches can be more rapidly exploited across a broader range of substrates or mutant proteins for engineering efforts. One area where this approach has been usefully exploited has been by calculating radical stabilities. Radical stabilities can be significantly modified by the encapsulating protein environment.^81−83^ An initial assessment of the influence of structural variation can be made through minimal active site models. Selected ab initio and/or DFT calculations are then carried out on a subset of key residues supported by crystallographic studies, and an assessment of accessible and tractable substrates and intermediates can be made. This approach was exemplified for *B. multivorans* QueE, a radical SAM enzyme,^81^ and laid the groundwork for benchmarking of a rapid and transferrable semiempirical-based workflow that could be applied across a broad range of substrate and protein-structural modifications.^82^ A thermodynamic reaction profile can be extracted from statistical analysis of computationally accessible MD simulations, followed by a triage of relevant substrate structures with semiempirical approaches. Subsequent QM single-point calculations provide the radical stabilization energies (RSEs) that inform the reaction profile and account for the impact of both protein structure and intermolecular interactions.

The electrostatic environment created by the protein also provides a key role in mediating cofactor/cosubstrate reactivity. The impact of changing the electrostatic environment on the reactivity of biologically relevant iron–sulfur clusters has been demonstrated with systematic quantum chemical assessment of a rotating static electric field represented by point-charges.^84^ Similarly, glycyl radical stabilization, important for the catalytic activity of PFL,^85^ responds to changes in electric-field orientation.^86^ Thus, modification of protein-induced electric fields through creating specific mutants, which may avoid changes to the coordination sphere of an active or cofactor site, offers future engineering routes for improved and designed reaction outcomes of radical reactions.

Taking the above summarized variables together, a transferable simulation and in silico screening workflow, including all those aspects and as presented in Figure 8, offers promising applicability for future rational radical enzyme design strategies. Starting by including the available diversity information of the specific enzyme target by bioinformatic approaches, the workflow could pass enzyme variants into a combined MD, QM/MM assessment pipeline to select promising mutagenesis suggestions that are then passed onto experimental assessment and validation. Within the pipeline, the strategy would include alternative substrate screening with rapid thermodynamic reaction profile assessment, which screens mutants for their effect on the thermodynamic stability of key radical intermediates and assesses the effect of internal electric fields on reaction kinetics. The outcomes here would again be able to inform on new potential variants with a maximal effect on either reactivity or substrate specificity, which may be supported by machine-learning.

Such a workflow approach should be ideally an experimental–computational collaboration. Current computational limitations might include the time to generate models, although this is rapidly decreasing with new iterations of structure prediction software.^87^ Model accuracy is often a concern but is rapidly improving to be in line with experimental structure-determination accuracy and tends to be less impactful within the active site regions. Failures in prediction, identified through the experimental validation, simply point to the need to support with additional good-quality experiments and possibly reveal more challenging and exciting systems to understand.

## Manipulating the Architecture

A structural view reveals that many radical enzymes can be comprised of “modules”;^21,26,88^ this offers an interesting avenue for future engineering efforts with the construction of radically initiated “Frankenzymes” (enzymes built of different parts, as per Frankenstein’s monster^89^) to carry out desired transformations once the underpinning catalytic features are understood in detail. Nature has already demonstrated this modular construction with some of the newly structurally characterized B_12_-AdoMet radical enzymes, which have discrete but integrated motifs to carry out dual chemistries.^10,11^ Similarly, RiPP enzymes possess a recognition element that can be used to guide a defined leader sequence to different active sites for transformation.^90^ Driven by the high reactivity of the initial Ado· radicals and subsequent substrate promiscuity that this can entail,^91^ a huge range of possibilities for new types of transformation is opened up if different active sites can be paired with different recognition elements in a modular fashion.

An alternative to working from larger, pre-existing modules is to start from the ground up, defining minimal sequences to carry out the chemistry required and incorporating these motifs as functional units in larger proteins. Minimal peptide motifs (in the form of maquettes), required to facilitate specific elements of some radical chemistries, have been successfully created. Protein-based radicals have been explored as potential radical chemistry initiators, in de novo designed maquettes, generating tyrosine and tryptophan radicals.^92^ In motifs corresponding to those found in ferrodoxin and radical SAM proteins,^93^ reconstitution of redox-active iron–sulfur clusters was shown to be in the range of 80–100%, although the conversion to the active form to initiate radical formation was lower, around 7–17% measured by EPR. De novo design tools, such as Rosetta^94^ and Omegafold,^87^ now allow structured peptides to be rapidly prototyped in silico, which can help to locate radical generating elements in 3D space for maximum effect, provide protective scaffolding for the radical, and enable integration into broader structures for future designs.

Combining the details available both from experimental work and bioinformatic studies, there are now real possibilities to design in radical chemistry to new proteins, retaining key features for specific chemistries/binding and using predictive approaches and rapid structural information provided by, *e.g.*, Alphafold and Alphafill,^55,56,95^ and other advanced machine-learning methods to derive the remainder of the protein.^87,96−99^ This has the potential to open up a huge new variety in the potential targets for chemical reaction and a better understanding of the key details of how these enzymes so carefully control these reactive intermediates.

## Future Outlook

There are a number of emerging radical enzyme candidates of potential industrial interest for which the mechanistic data required for rational design is now available. Such enzymes include phenylacetate decarboxylase (PhdB), a glycyl-radical enzyme that is able to produce toluene from renewable resources;^100^ the huge range of AdoMet radical enzymes involved in ribosomally synthesized and post-translationally modified peptide (RiPP) biosynthesis pathways, which offer scope for new antimicrobials among other interesting activities;^7,8,101^ AdoMet enzymes such as the sulfatase AtsB, which can be utilized to create labeled peptides;^102^ and many others that may be involved in, for example, environmental detoxification.^67,103^

A critical combination of high-quality structural data, insightful kinetic experiments, and computational approaches is essential to bring these enzymes into new niches in the industrial domain. Although challenging because of both the reactivity and open-shell nature of the intermediates for these enzymes, inroads are being made, with the unique chemistries catalyzed being an attractive proposition. The bringing together of high-throughput approaches signifies an opening to rapidly realizing new, designed modifications,^98^ provided they are integrated with the lessons already learned for these amazing radical enzymes.

## References

[ref1] Bryan M. C.; Dunn P. J.; Entwistle D.; Gallou F.; Koenig S. G.; Hayler J. D.; Hickey M. R.; Hughes S.; Kopach M. E.; Moine G.; Richardson P.; Roschangar F.; Steven A.; Weiberth F. J. Key Green Chemistry research areas from a pharmaceutical manufacturers’ perspective revisited. Green Chem. 2018, 20, 5082–5103. 10.1039/C8GC01276H.

[ref2] Constable D. J. C.; Dunn P. J.; Hayler J. D.; Humphrey G. R.; Leazer J. L. Jr.; Linderman R. J.; Lorenz K.; Manley J.; Pearlman B. A.; Wells A.; Zaks A.; Zhang T. Y. Key green chemistry research areas—a perspective from pharmaceutical manufacturers. Green Chem. 2007, 9, 411–420. 10.1039/B703488C.

[ref3] Fu B.; Balskus E. P. Discovery of CC bond-forming and bond-breaking radical enzymes: enabling transformations for metabolic engineering. Curr. Opin. Biotechnol. 2020, 65, 94–101. 10.1016/j.copbio.2020.02.003. 32171888 PMC7670169

[ref4] Yokoyama K.; Lilla E. A. C-C bond forming radical SAM enzymes involved in the construction of carbon skeletons of cofactors and natural products. Nat. Prod. Rep. 2018, 35, 660–694. 10.1039/C8NP00006A. 29633774 PMC6051890

[ref5] Croft A. K.; Lindsay K. B.; Renaud P.; Skrydstrup T. Radicals by Design. Chimia 2008, 62, 735–741. 10.2533/chimia.2008.735.

[ref6] Bandarian V. Radical SAM enzymes involved in the biosynthesis of purine-based natural products. Biochim. Biophys. Acta 2012, 1824, 1245–1253. 10.1016/j.bbapap.2012.07.014. 22902275 PMC4022190

[ref7] Lewis J. K.; Jochimsen A. S.; Lefave S. J.; Young A. P.; Kincannon W. M.; Roberts A. G.; Kieber-Emmons M. T.; Bandarian V. New Role for Radical SAM Enzymes in the Biosynthesis of Thio(seleno)oxazole RiPP Natural Products. Biochemistry 2021, 60, 3347–3361. 10.1021/acs.biochem.1c00469. 34730336 PMC8935624

[ref8] Montalban-Lopez M.; Scott T. A.; Ramesh S.; Rahman I. R.; van Heel A. J.; Viel J. H.; Bandarian V.; Dittmann E.; Genilloud O.; Goto Y.; Grande Burgos M. J.; Hill C.; Kim S.; Koehnke J.; Latham J. A.; Link A. J.; Martinez B.; Nair S. K.; Nicolet Y.; Rebuffat S.; Sahl H. G.; Sareen D.; Schmidt E. W.; Schmitt L.; Severinov K.; Sussmuth R. D.; Truman A. W.; Wang H.; Weng J. K.; van Wezel G. P.; Zhang Q.; Zhong J.; Piel J.; Mitchell D. A.; Kuipers O. P.; van der Donk W. A. New developments in RiPP discovery, enzymology and engineering. Nat. Prod. Rep. 2021, 38, 130–239. 10.1039/D0NP00027B. 32935693 PMC7864896

[ref9] McCarty R. M.; Bandarian V. Biosynthesis of pyrrolopyrimidines. Bioorg. Chem. 2012, 43, 15–25. 10.1016/j.bioorg.2012.01.001. 22382038 PMC4022189

[ref10] Bridwell-Rabb J.; Zhong A.; Sun H. G.; Drennan C. L.; Liu H. W. A B12-dependent radical SAM enzyme involved in oxetanocin A biosynthesis. Nature 2017, 544, 322–326. 10.1038/nature21689. 28346939 PMC5398914

[ref11] Knox H. L.; Sinner E. K.; Townsend C. A.; Boal A. K.; Booker S. J. Structure of a B12-dependent radical SAM enzyme in carbapenem biosynthesis. Nature 2022, 602, 343–348. 10.1038/s41586-021-04392-4. 35110734 PMC8950224

[ref12] Buckel W.; Golding B. T. Radical enzymes in anaerobes. Annu. Rev. Microbiol. 2006, 60, 27–49. 10.1146/annurev.micro.60.080805.142216. 16704345

[ref13] Shibata N.; Toraya T. Molecular architectures and functions of radical enzymes and their (re)activating proteins. J. Biochem. 2015, 158, 271–292. 10.1093/jb/mvv078. 26261050

[ref14] Salaemae W.; Azhar A.; Booker G. W.; Polyak S. W. Biotin biosynthesis in Mycobacterium tuberculosis: physiology, biochemistry and molecular intervention. Protein Cell 2011, 2, 691–695. 10.1007/s13238-011-1100-8. 21976058 PMC4875270

[ref15] Peck S. C.; Denger K.; Burrichter A.; Irwin S. M.; Balskus E. P.; Schleheck D. A glycyl radical enzyme enables hydrogen sulfide production by the human intestinal bacterium Bilophila wadsworthia. Proc. Natl. Acad. Sci. U.S.A 2019, 116, 3171–3176. 10.1073/pnas.1815661116. 30718429 PMC6386719

[ref16] Levin B. J.; Huang Y. Y.; Peck S. C.; Wei Y.; Martinez-Del Campo A.; Marks J. A.; Franzosa E. A.; Huttenhower C.; Balskus E. P. A prominent glycyl radical enzyme in human gut microbiomes metabolizes trans-4-hydroxy-l-proline. Science 2017, 355, eaai8386 10.1126/science.aai8386. 28183913 PMC5705181

[ref17] Levin B. J.; Balskus E. P. Discovering radical-dependent enzymes in the human gut microbiota. Curr. Opin. Chem. Biol. 2018, 47, 86–93. 10.1016/j.cbpa.2018.09.011. 30268905

[ref18] Wei Y.; Zhang Y. Glycyl Radical Enzymes and Sulfonate Metabolism in the Microbiome. Annu. Rev. Biochem. 2021, 90, 817–846. 10.1146/annurev-biochem-080120-024103. 33823652

[ref19] Landgraf B. J.; McCarthy E. L.; Booker S. J. Radical S-Adenosylmethionine Enzymes in Human Health and Disease. Annu. Rev. Biochem. 2016, 85, 485–514. 10.1146/annurev-biochem-060713-035504. 27145839

[ref20] Jäger C. M.; Croft A. K. Anaerobic Radical Enzymes for Biotechnology. ChemBioEng. Rev. 2018, 5, 143–162. 10.1002/cben.201800003.

[ref21] Dowling D. P.; Croft A. K.; Drennan C. L. Radical use of Rossmann and TIM barrel architectures for controlling coenzyme B12 chemistry. Annu. Rev. Biophys. 2012, 41, 403–427. 10.1146/annurev-biophys-050511-102225. 22577824

[ref22] Cracan V.; Banerjee R. Novel B(12)-dependent acyl-CoA mutases and their biotechnological potential. Biochemistry 2012, 51, 6039–6046. 10.1021/bi300827v. 22803641 PMC3448797

[ref23] Bridwell-Rabb J.; Grell T. A. J.; Drennan C. L. A Rich Man, Poor Man Story of S-Adenosylmethionine and Cobalamin Revisited. Annu. Rev. Biochem. 2018, 87, 555–584. 10.1146/annurev-biochem-062917-012500. 29925255

[ref24] Broderick J. B.; Duffus B. R.; Duschene K. S.; Shepard E. M. Radical S-adenosylmethionine enzymes. Chem. Rev. 2014, 114, 4229–4317. 10.1021/cr4004709. 24476342 PMC4002137

[ref25] Babbitt Lab SFLD Team. Structure Function Linkage Database, Radical SAM Superfamily, http://sfld.rbvi.ucsf.edu/archive/django/superfamily/29/index.html.

[ref26] Holliday G. L.; Akiva E.; Meng E. C.; Brown S. D.; Calhoun S.; Pieper U.; Sali A.; Booker S. J.; Babbitt P. C. Atlas of the Radical SAM Superfamily: Divergent Evolution of Function Using a ″Plug and Play″ Domain. Methods Enzymol. 2018, 606, 1–71. 10.1016/bs.mie.2018.06.004. 30097089 PMC6445391

[ref27] Shisler K. A.; Broderick J. B. Glycyl radical activating enzymes: structure, mechanism, and substrate interactions. Arch. Biochem. Biophys. 2014, 546, 64–71. 10.1016/j.abb.2014.01.020. 24486374 PMC4083501

[ref28] Backman L. R. F.; Funk M. A.; Dawson C. D.; Drennan C. L. New tricks for the glycyl radical enzyme family. Crit. Rev. Biochem. Mol. Biol. 2017, 52, 674–695. 10.1080/10409238.2017.1373741. 28901199 PMC5911432

[ref29] Selmer T.; Pierik A. J.; Heider J. New glycyl radical enzymes catalysing key metabolic steps in anaerobic bacteria. Biol. Chem. 2005, 386, 981–988. 10.1515/BC.2005.114. 16218870

[ref30] Dowling D. P.; Vey J. L.; Croft A. K.; Drennan C. L. Structural diversity in the AdoMet radical enzyme superfamily. Biochim. Biophys. Acta 2012, 1824, 1178–1195. 10.1016/j.bbapap.2012.04.006. 22579873 PMC3523193

[ref31] Buckel W. Enzymatic Reactions Involving Ketyls: From a Chemical Curiosity to a General Biochemical Mechanism. Biochemistry 2019, 58, 5221–5233. 10.1021/acs.biochem.9b00171. 30995029

[ref32] Zhang Y.; Liu D.; Chen Z. Production of C2-C4 diols from renewable bioresources: new metabolic pathways and metabolic engineering strategies. Biotechnol. Biofuels 2017, 10, 299 10.1186/s13068-017-0992-9. 29255482 PMC5727944

[ref33] Nicolet Y. Structure–function relationships of radical SAM enzymes. Nature Catal 2020, 3, 337–350. 10.1038/s41929-020-0448-7.

[ref34] Nakamura C. E.; Whited G. M. Metabolic engineering for the microbial production of 1,3-propanediol. Curr. Opin. Biotechnol. 2003, 14, 454–459. 10.1016/j.copbio.2003.08.005. 14580573

[ref35] Yamanishi M.; Kinoshita K.; Fukuoka M.; Saito T.; Tanokuchi A.; Ikeda Y.; Obayashi H.; Mori K.; Shibata N.; Tobimatsu T.; Toraya T. Redesign of coenzyme B(12) dependent diol dehydratase to be resistant to the mechanism-based inactivation by glycerol and act on longer chain 1,2-diols. FEBS J. 2012, 279, 793–804. 10.1111/j.1742-4658.2012.08470.x. 22221669

[ref36] Wang J.; Jain R.; Shen X.; Sun X.; Cheng M.; Liao J. C.; Yuan Q.; Yan Y. Rational engineering of diol dehydratase enables 1,4-butanediol biosynthesis from xylose. Metab. Eng. 2017, 40, 148–156. 10.1016/j.ymben.2017.02.003. 28215518

[ref37] Bachovchin W. W.; Moore K. W.; Richards J. H. Mechanism of action of adenosylcobalamin: hydrogen transfer in the inactivation of diol dehydratase by glycerol. Biochemistry 1978, 17, 2218–2224. 10.1021/bi00604a031. 667021

[ref38] Bilic L.; Baric D.; Banhatti R. D.; Smith D. M.; Kovacevic B. Computational Study of Glycerol Binding within the Active Site of Coenzyme B12-Dependent Diol Dehydratase. J. Phys. Chem. B 2019, 123, 6178–6187. 10.1021/acs.jpcb.9b04071. 31251060

[ref39] Bilic L.; Baric D.; Sandala G. M.; Smith D. M.; Kovacevic B. Glycerol as a Substrate and Inactivator of Coenzyme B12 -Dependent Diol Dehydratase. Chem. Eur. J. 2021, 27, 7930–7941. 10.1002/chem.202100416. 33792120

[ref40] Jain R.; Sun X.; Yuan Q.; Yan Y. Systematically engineering Escherichia coli for enhanced production of 1,2-propanediol and 1-propanol. ACS Synth. Biol. 2015, 4, 746–756. 10.1021/sb500345t. 25490349

[ref41] Gibson K. J.; Liao D.-I.; Tang X.-S. B12-dependent dehydratases with improved reaction kinetics, US8445659B2, E I Du Pont de Nemours and Company, US, 2010.

[ref42] Chandor-Proust A.; Berteau O.; Douki T.; Gasparutto D.; Ollagnier-de-Choudens S.; Fontecave M.; Atta M. DNA repair and free radicals, new insights into the mechanism of spore photoproduct lyase revealed by single amino acid substitution. J. Biol. Chem. 2008, 283, 36361–36368. 10.1074/jbc.M806503200. 18957420 PMC2662300

[ref43] Benjdia A.; Heil K.; Barends T. R.; Carell T.; Schlichting I. Structural insights into recognition and repair of UV-DNA damage by Spore Photoproduct Lyase, a radical SAM enzyme. Nucleic Acids Res. 2012, 40, 9308–9318. 10.1093/nar/gks603. 22761404 PMC3467042

[ref44] Yang L.; Lin G.; Nelson R. S.; Jian Y.; Telser J.; Li L. Mechanistic studies of the spore photoproduct lyase via a single cysteine mutation. Biochemistry 2012, 51, 7173–7188. 10.1021/bi3010945. 22906093 PMC3448869

[ref45] Yang L.; Nelson R. S.; Benjdia A.; Lin G.; Telser J.; Stoll S.; Schlichting I.; Li L. A radical transfer pathway in spore photoproduct lyase. Biochemistry 2013, 52, 3041–3050. 10.1021/bi3016247. 23607538 PMC3666868

[ref46] Ji X.; Li Y.; Ding W.; Zhang Q. Substrate-Tuned Catalysis of the Radical S-Adenosyl-L-Methionine Enzyme NosL Involved in Nosiheptide Biosynthesis. Angew. Chem., Int. Ed. Engl. 2015, 54, 9021–9024. 10.1002/anie.201503976. 26138750

[ref47] Ji X.; Li Y.; Jia Y.; Ding W.; Zhang Q. Mechanistic Insights into the Radical S-adenosyl-l-methionine Enzyme NosL From a Substrate Analogue and the Shunt Products. Angew. Chem., Int. Ed. Engl. 2016, 55, 3334–3337. 10.1002/anie.201509900. 26837062

[ref48] Bhandari D. M.; Xu H.; Nicolet Y.; Fontecilla-Camps J. C.; Begley T. P. Tryptophan Lyase (NosL): Mechanistic Insights from Substrate Analogues and Mutagenesis. Biochemistry 2015, 54, 4767–4769. 10.1021/acs.biochem.5b00764. 26204056

[ref49] Ji X.; Li Y.; Xie L.; Lu H.; Ding W.; Zhang Q. Expanding Radical SAM Chemistry by Using Radical Addition Reactions and SAM Analogues. Angew. Chem., Int. Ed. Engl. 2016, 55, 11845–11848. 10.1002/anie.201605917. 27573794

[ref50] Ding W.; Ji X.; Li Y.; Zhang Q. Catalytic Promiscuity of the Radical S-adenosyl-L-methionine Enzyme NosL. Front. Chem. 2016, 4, 27 10.3389/fchem.2016.00027. 27446906 PMC4916742

[ref51] Bhandari D. M.; Fedoseyenko D.; Begley T. P. Tryptophan Lyase (NosL): A Cornucopia of 5′-Deoxyadenosyl Radical Mediated Transformations. J. Am. Chem. Soc. 2016, 138, 16184–16187. 10.1021/jacs.6b06139. 27998091

[ref52] Nicolet Y.; Zeppieri L.; Amara P.; Fontecilla-Camps J. C. Crystal structure of tryptophan lyase (NosL): evidence for radical formation at the amino group of tryptophan. Angew. Chem., Int. Ed. Engl. 2014, 53, 11840–11844. 10.1002/anie.201407320. 25196319

[ref53] Liao H. H.; Gokarn R. R.; Gort S. J.; Jessen H. J.; Selifonova O. Alanine 2,3-Aminomutase, US7309597B2, Cargill Inc, 2007.

[ref54] Spadoni D. Computational investigation on the mechanism of oxygen tolerance in the radical SAM enzyme lysine 2,3-aminomutase. Ph.D. Thesis, University of Nottingham, Nottingham, 2022.

[ref55] Jumper J.; Evans R.; Pritzel A.; Green T.; Figurnov M.; Ronneberger O.; Tunyasuvunakool K.; Bates R.; Zidek A.; Potapenko A.; Bridgland A.; Meyer C.; Kohl S. A. A.; Ballard A. J.; Cowie A.; Romera-Paredes B.; Nikolov S.; Jain R.; Adler J.; Back T.; Petersen S.; Reiman D.; Clancy E.; Zielinski M.; Steinegger M.; Pacholska M.; Berghammer T.; Bodenstein S.; Silver D.; Vinyals O.; Senior A. W.; Kavukcuoglu K.; Kohli P.; Hassabis D. Highly accurate protein structure prediction with AlphaFold. Nature 2021, 596, 583–589. 10.1038/s41586-021-03819-2. 34265844 PMC8371605

[ref56] Varadi M.; Anyango S.; Deshpande M.; Nair S.; Natassia C.; Yordanova G.; Yuan D.; Stroe O.; Wood G.; Laydon A.; Zidek A.; Green T.; Tunyasuvunakool K.; Petersen S.; Jumper J.; Clancy E.; Green R.; Vora A.; Lutfi M.; Figurnov M.; Cowie A.; Hobbs N.; Kohli P.; Kleywegt G.; Birney E.; Hassabis D.; Velankar S. AlphaFold Protein Structure Database: massively expanding the structural coverage of protein-sequence space with high-accuracy models. Nucleic Acids Res. 2022, 50, D439–D444. 10.1093/nar/gkab1061. 34791371 PMC8728224

[ref57] Sharma P. K.; Chu Z. T.; Olsson M. H.; Warshel A. A new paradigm for electrostatic catalysis of radical reactions in vitamin B12 enzymes. Proc. Natl. Acad. Sci. U.S.A 2007, 104, 9661–9666. 10.1073/pnas.0702238104. 17517615 PMC1887576

[ref58] Kirst H.; Ferlez B. H.; Lindner S. N.; Cotton C. A. R.; Bar-Even A.; Kerfeld C. A. Toward a glycyl radical enzyme containing synthetic bacterial microcompartment to produce pyruvate from formate and acetate. Proc. Natl. Acad. Sci. U.S.A. 2022, 119, e2116871119 10.1073/pnas.2116871119. 35193962 PMC8872734

[ref59] Knappe J.; Sawers G. A radical-chemical route to acetyl-CoA: the anaerobically induced pyruvate formate-lyase system of *Escherichia coli*. FEMS Microbiol. Rev. 1990, 75, 383–398. 10.1111/j.1574-6968.1990.tb04108.x. 2248795

[ref60] Crain A. V.; Broderick J. B. Pyruvate formate-lyase and its activation by pyruvate formate-lyase activating enzyme. J. Biol. Chem. 2014, 289, 5723–5729. 10.1074/jbc.M113.496877. 24338017 PMC3937645

[ref61] Hanzevacki M.; Banhatti R. D.; Condic-Jurkic K.; Smith A. S.; Smith D. M. Exploring Reactive Conformations of Coenzyme A during Binding and Unbinding to Pyruvate Formate-Lyase. J. Phys. Chem. A 2019, 123, 9345–9356. 10.1021/acs.jpca.9b06913. 31580071

[ref62] Hanzevacki M.; Condic-Jurkic K.; Banhatti R. D.; Smith A. S.; Smith D. M. The Influence of Chemical Change on Protein Dynamics: A Case Study with Pyruvate Formate-Lyase. Chem. Eur. J. 2019, 25, 8741–8753. 10.1002/chem.201900663. 30901109

[ref63] Ferlez B.; Sutter M.; Kerfeld C. A. Glycyl Radical Enzyme-Associated Microcompartments: Redox-Replete Bacterial Organelles. mBio 2019, 10, e02327-18 10.1128/mBio.02327-18. 30622187 PMC6325248

[ref64] Zarzycki J.; Erbilgin O.; Kerfeld C. A. Bioinformatic characterization of glycyl radical enzyme-associated bacterial microcompartments. Appl. Environ. Microbiol. 2015, 81, 8315–8329. 10.1128/AEM.02587-15. 26407889 PMC4644659

[ref65] Bali A. P.; Lennox-Hvenekilde D.; Myling-Petersen N.; Buerger J.; Salomonsen B.; Gronenberg L. S.; Sommer M. O. A.; Genee H. J. Improved biotin, thiamine, and lipoic acid biosynthesis by engineering the global regulator IscR. Metab. Eng. 2020, 60, 97–109. 10.1016/j.ymben.2020.03.005. 32220614

[ref66] Xiao F.; Wang H.; Shi Z.; Huang Q.; Huang L.; Lian J.; Cai J.; Xu Z. Multi-level metabolic engineering of Pseudomonas mutabilis ATCC31014 for efficient production of biotin. Metab. Eng. 2020, 61, 406–415. 10.1016/j.ymben.2019.05.005. 31085296

[ref67] Wang Y.; Nguyen N.; Lee S. H.; Wang Q.; May J. A.; Gonzalez R.; Cirino P. C. Engineering Escherichia coli for anaerobic alkane activation: Biosynthesis of (1-methylalkyl)succinates. Biotechnol. Bioeng. 2022, 119, 315–320. 10.1002/bit.27956. 34633065

[ref68] Akhtar M. K.; Jones P. R. Deletion of iscR stimulates recombinant clostridial Fe-Fe hydrogenase activity and H2-accumulation in Escherichia coli BL21(DE3). Appl. Microbiol. Biotechnol. 2008, 78, 853–862. 10.1007/s00253-008-1377-6. 18320190

[ref69] Kuchenreuther J. M.; Grady-Smith C. S.; Bingham A. S.; George S. J.; Cramer S. P.; Swartz J. R. High-yield expression of heterologous [FeFe] hydrogenases in Escherichia coli. PLoS One 2010, 5, e15491 10.1371/journal.pone.0015491. 21124800 PMC2991362

[ref70] Blue T. C.; Davis K. M. Computational Approaches: An Underutilized Tool in the Quest to Elucidate Radical SAM Dynamics. Molecules 2021, 26, 2590 10.3390/molecules26092590. 33946806 PMC8124187

[ref71] Kovacevic B.; Baric D.; Babic D.; Bilic L.; Hanzevacki M.; Sandala G. M.; Radom L.; Smith D. M. Computational Tale of Two Enzymes: Glycerol Dehydration With or Without B12. J. Am. Chem. Soc. 2018, 140, 8487–8496. 10.1021/jacs.8b03109. 29894625

[ref72] Sandala G. M.; Smith D. M.; Radom L. Modeling the Reactions Catalyzed by Coenzyme B12-Dependent Enzymes. Acc. Chem. Res. 2010, 43, 642–651. 10.1021/ar900260c. 20136160

[ref73] Toda M. J.; Ghosh A. P.; Parmar S.; Kozlowski P. M. Computational investigations of B12-dependent enzymatic reactions. Methods Enzymol. 2022, 669, 119–150. 10.1016/bs.mie.2022.01.002. 35644169

[ref74] Liu Y.; Gallo A. A.; Florián J.; Liu Y.-S.; Mora S.; Xu W. QM/MM (ONIOM) Study of Glycerol Binding and Hydrogen Abstraction by the Coenzyme B_12_-Independent Dehydratase. J. Phys. Chem. B 2010, 114, 5497–5502. 10.1021/jp910349q. 20361776

[ref75] Zhu W.; Liu Y. Ring Contraction Catalyzed by the Metal-Dependent Radical SAM Enzyme: 7-Carboxy-7-deazaguanine Synthase from B. multivorans. Theoretical Insights into the Reaction Mechanism and the Influence of Metal Ions. ACS Catal. 2015, 5, 3953–3965. 10.1021/acscatal.5b00156.

[ref76] Amara P.; Saragaglia C.; Mouesca J. M.; Martin L.; Nicolet Y. L-tyrosine-bound ThiH structure reveals C-C bond break differences within radical SAM aromatic amino acid lyases. Nat. Commun. 2022, 13, 2284 10.1038/s41467-022-29980-4. 35477710 PMC9046217

[ref77] Zhu W.; Liu Y.; Zhang R. QM/MM study of the conversion mechanism of lysine to methylornithine catalyzed by methylornithine synthase (PylB). Theor. Chem. Acc. 2013, 132, 1385 10.1007/s00214-013-1385-1.

[ref78] Zhou S.; Wei W.-J.; Liao R.-Z. QM/MM Study of the Mechanism of the Noncanonical S-Cγ Bond Scission in S-Adenosylmethionine Catalyzed by the CmnDph2 Radical Enzyme. Topics Catal. 2022, 65, 517–527. 10.1007/s11244-021-01420-5.

[ref79] Zhao C.; Dong L.; Liu Y. A QM/MM study of the catalytic mechanism of SAM methyltransferase RlmN from Escherichia coli. Proteins 2017, 85, 1967–1974. 10.1002/prot.25337. 28643349

[ref80] Yan L.; Liu Y. Mechanistic Insights into the Anaerobic Degradation of Globally Abundant Dihydroxypropanesulfonate Catalyzed by the DHPS-Sulfolyase (HpsG). J. Chem. Inf. Model. 2022, 62, 2880–2888. 10.1021/acs.jcim.2c00174. 35583151

[ref81] Jäger C. M.; Croft A. K. Radical Reaction Control in the AdoMet Radical Enzyme CDG Synthase (QueE): Consolidate, Destabilize, Accelerate. Chem. Eur. J. 2017, 23, 953–962. 10.1002/chem.201604719. 27859789 PMC5347944

[ref82] Suess C. J.; Martins F. L.; Croft A. K.; Jäger C. M. Radical Stabilization Energies for Enzyme Engineering: Tackling the Substrate Scope of the Radical Enzyme QueE. J. Chem. Inf. Model. 2019, 59, 5111–5125. 10.1021/acs.jcim.9b00017. 31730347

[ref83] Hanževački M.; Croft A. K.; Jäger C. M. Activation of Glycyl Radical Enzymes – Multiscale Modeling Insights into Catalysis and Radical Control in Pyruvate Formate-Lyase Activating Enzyme. J. Chem. Inf. Model. 2022, 62, 3401 10.1021/acs.jcim.2c00362. 35771966 PMC9326890

[ref84] Gaughan S. J. H.; Hirst J. D.; Croft A. K.; Jäger C. M. Effect of Oriented Electric Fields on Biologically Relevant Iron-Sulfur Clusters: Tuning Redox Reactivity for Catalysis. J. Chem. Inf. Model. 2022, 62, 591–601. 10.1021/acs.jcim.1c00791. 35045248

[ref85] Hioe J.; Savasci G.; Brand H.; Zipse H. The stability of C_α_ peptide radicals: why glycyl radical enzymes?. Chem. Eur. J. 2011, 17, 3781–3789. 10.1002/chem.201002620. 21341321

[ref86] Jangra H.; Zipse H. Electrostatic Effects on the Stability of Peptide Radicals. J. Phys. Chem. B 2018, 122, 8880–8890. 10.1021/acs.jpcb.8b07485. 30199247

[ref87] Wu R.; Ding F.; Wang R.; Shen R.; Zhang X.; Luo S.; Su C.; Wu Z.; Xie Q.; Berger B.; Ma J.; Peng J. High-resolution de novo structure prediction from primary sequence. BioRxiv 2022, 10.1101/2022.07.21.500999.

[ref88] Grell T. A.; Goldman P. J.; Drennan C. L. SPASM and twitch domains in S-adenosylmethionine (SAM) radical enzymes. J. Biol. Chem. 2015, 290, 3964–3971. 10.1074/jbc.R114.581249. 25477505 PMC4326806

[ref89] Shelley M. Frankenstein: Annotated for scientists, engineers and creators of all kinds; Guston D. H., Finn E., Robert J. S., Eds.;The MIT Press, 2017.

[ref90] Burkhart B. J.; Hudson G. A.; Dunbar K. L.; Mitchell D. A. A prevalent peptide-binding domain guides ribosomal natural product biosynthesis. Nat. Chem. Biol. 2015, 11, 564–570. 10.1038/nchembio.1856. 26167873 PMC4509860

[ref91] Eastman K. A. S.; Kincannon W. M.; Bandarian V. Leveraging Substrate Promiscuity of a Radical S-Adenosyl-L-methionine RiPP Maturase toward Intramolecular Peptide Cross-Linking Applications. ACS Cent. Sci. 2022, 8, 1209 10.1021/acscentsci.2c00501. 36032765 PMC9413430

[ref92] Tommos C.; Skalicky J. J.; Pilloud D. L.; Wand A. J.; Dutton P. L. De novo proteins as models of radical enzymes. Biochemistry 1999, 38, 9495 10.1021/bi990609g. 10413527

[ref93] Galambas A.; Miller J.; Jones M.; McDaniel E.; Lukes M.; Watts H.; Copie V.; Broderick J. B.; Szilagyi R. K.; Shepard E. M. Radical S-adenosylmethionine maquette chemistry: Cx3Cx2C peptide coordinated redox active [4Fe-4S] clusters. J. Biol. Inorg. Chem. 2019, 24, 793–807. 10.1007/s00775-019-01708-8. 31486952

[ref94] Baker D. Rosetta Commons. https://www.rosettacommons.org/software.

[ref95] Hekkelman M. L.; de Vries I.; Joosten R. P.; Perrakis A. AlphaFill: enriching the AlphaFold models with ligands and co-factors. BioRxiv 2021, 10.1101/2021.11.26.470110. PMC9911346 36424442

[ref96] Dauparas J.; Anishchenko I.; Bennett N.; Bai H.; Ragotte R. J.; Milles L. F.; Wicky B. I. M.; Courbet A.; de Haas R. J.; Bethel N.; Leung P. J. Y.; Huddy T. F.; Pellock S.; Tischer D.; Chan F.; Koepnick B.; Nguyen H.; Kang A.; Sankaran B.; Bera A.; King N. P.; Baker D. Robust deep learning based protein sequence design using ProteinMPNN. BioRXiv 2022, 10.1101/2022.06.03.494563. PMC9997061 36108050

[ref97] Anand N.; Achim T. Protein Structure and Sequence Generation with Equivariant Denoising Diffusion Probabilistic Models. arXiv 2022, arXiv:2205.15019v15011 10.48550/arXiv.2205.15019.

[ref98] Lovelock S. L.; Crawshaw R.; Basler S.; Levy C.; Baker D.; Hilvert D.; Green A. P. The road to fully programmable protein catalysis. Nature 2022, 606, 49–58. 10.1038/s41586-022-04456-z. 35650353

[ref99] Trippe B. L.; Yim J.; Tischer D.; Baker D.; Broderick T.; Barzilay R.; Jaakkola T. Diffusion probabilistic modeling of protein backbones in 3D for the motif-scaffolding problem. arXiv 2022, arXiv:2206.04119v04111 10.48550/arXiv.2206.04119.

[ref100] Rodrigues A. V.; Tantillo D. J.; Mukhopadhyay A.; Keasling J. D.; Beller H. R. Insight into the Mechanism of Phenylacetate Decarboxylase (PhdB), a Toluene-Producing Glycyl Radical Enzyme. Chembiochem 2020, 21, 663–671. 10.1002/cbic.201900560. 31512343 PMC7079210

[ref101] Benjdia A.; Berteau O. Radical SAM Enzymes and Ribosomally-Synthesized and Post-translationally Modified Peptides: A Growing Importance in the Microbiomes. Front. Chem. 2021, 9, 678068 10.3389/fchem.2021.678068. 34350157 PMC8326336

[ref102] Kruger T.; Weiland S.; Boschanski M.; Sinha P. K.; Falck G.; Muller K. M.; Dierks T.; Sewald N. Conversion of Serine-Type Aldehyde Tags by the Radical SAM Protein AtsB from Methanosarcina mazei. Chembiochem 2019, 20, 2074–2078. 10.1002/cbic.201900322. 31215729

[ref103] Boll M.; Estelmann S.; Heider J. Anaerobic Degradation of Hydrocarbons: Mechanisms of Hydrocarbon Activation in the Absence of Oxygen. Anaerobic Utilization of Hydrocarbons, Oils, and Lipids 2020, 3–29. 10.1007/978-3-319-50391-2_2.

